# Impact of Polystyrene Micro- and Nanoplastics on the Biological Traits of the Japanese Carpenter Ant, *Camponotus japonicus* Mayr (Hymenoptera: Formicidae)

**DOI:** 10.3390/insects16030292

**Published:** 2025-03-11

**Authors:** Li-Feng Wei, Xin-Ying Liu, Han-Song Feng, Jiang-Tao Zhang, Xing-Ping Liu

**Affiliations:** Provincial Key Laboratory of Conservation Biology, School of Forestry, Jiangxi Agricultural University, Nanchang 330045, China; lifengwei102@163.com (L.-F.W.); 15912934033@163.com (X.-Y.L.); 13576800477@163.com (H.-S.F.); jiang_tao_zhang@163.com (J.-T.Z.)

**Keywords:** *Camponotus japonicus*, polystyrene, microplastics, nanoplastics, foraging preference, biological traits

## Abstract

Insects, being among the most diverse and abundant organisms in terrestrial ecosystems, are inevitably exposed to ubiquitous micro- and nanoplastic contaminants. Although studies on the impact of these contaminants on terrestrial insects are gradually emerging, they remain limited in scope. In this study, we investigated the biological traits (including foraging behavior, food assumption, digging ability, body weight and survival) of the Japanese carpenter ant, *Camponotus japonicus*, in response to exposure to polystyrene micro- and nanoplastic (PS-M/NP) solutions containing three particle sizes (0.05, 1 and 50 μm) and four concentrations (0.1, 1, 10 and 50 mg/mL). The results showed that worker ants exhibited significant foraging preference and food consumption for non-contaminated solutions in multiple-choice experiments, indicating that worker ants *C. japonicus* can differentiate and avoid feeding on PS-M/NP-contaminated solutions. Meanwhile, PS-M/NPs significantly reduced the foraging ability of worker ants in multiple-choice and no-choice experiments, with the smallest particle size (0.05 μm) and highest concentration (50 mg/mL) of PS-M/NPs resulting in the longest pre-foraging period, the lowest percentage of licking and the amount of food consumption. In addition, the weight of sand removed by worker ants, the body weight and survival of worker ants showed a dramatic decline with a decrease in particle size, increase in concentration and prolonged in exposure time of PS-M/NP-contaminated solutions. The results of this study confirm that PS-M/NPs have an adverse effect on these worker ants in a particle size, concentration and exposure time-dependent manner, with small particle size, high concentration and longer exposure time being the key factors in decreasing the foraging behavior and biological traits of this insect.

## Simple Summary

The transport and accumulation of ubiquitous micro- and nanoplastics (M/NPs) by ants make them a good object for studying the impact of M/NPs pollution on terrestrial insects because it is a prominent eusocial insect species in almost all terrestrial ecosystems worldwide. This study sought to investigate the impact of polystyrene micro- and nanoplastics (PS-M/NPs) on the biological traits in a laboratory setting utilizing the Japanese carpenter ant, *Camponotus japonicus*, as an experimental insect. We found that the worker ants showed the ability to distinguish between normal and PS-M/NP-contaminated solutions and chase non-contaminated solutions. In addition, PS-M/NPs had adverse effects on these worker ants in terms of foraging behavior, digging ability, body weight and survival. These adverse effects of PS-M/NPs were regulated by their particle size, concentration and exposure time. Our findings provide a baseline understanding of the effects of PS-M/NPs on this ant species and give a warning that management measures against PS-M/NPs must be taken.

## 1. Introduction

Many scientific studies have reported that nearly half of the world’s insect species are in rapid decline, and a third have been threatened for decades [1,2,3]. Globally, the well-known cause of insect decline has been attributed to many factors, including habitat loss, agricultural intensification, pollution, pathogens and biological invasions, and climate change [1,4,5]. Among these factors, plastic pollution, including microplastics (diameter ranging from 1 μm to 5 mm, hereafter MPs), nanoplastics (diameter smaller than 1 μm, hereafter NPs) and their associated polymers, is considered one of the important environmental pollutants contributing to insect decline and has received growing attention recently [1,3,6,7,8,9,10,11]. Micro- and nanoplastics (hereafter M/NPs) generated from plastic waste have been detected in nearly every environment on Earth, including air, plant, soil, water, sediment and other environmental matrices, a sign of their ubiquitous presence in human and wildlife surroundings [10,12]. These persistent particles in the environment may bioaccumulate in living organisms and, subsequently, biomagnification through trophic transfer from soil to producer to consumers, including humans [13,14,15]. Undoubtedly, as the most diverse and numerous groups of organisms in terrestrial ecosystems, insects are inevitably exposed to ubiquitous M/NPs [9,11,16,17].

The tiny size of M/NPs enables them to be easily disseminated and ingested by insects, which can lead to their accumulation within the food chain and ultimately produce certain adverse effects on the physiology, behavior and fitness of insects [18,19,20]. In addition, insects can also be entangled in plastics or die trapped in discarded plastic bottles and cans [21,22]. These adverse effects of M/NPs have been described in many insects, such as freshwater diving beetles [23], mosquitoes [18,24], dragonflies [25], fruit flies [26,27], honeybees [10,28,29], silkworms [30], greater wax moths [31], tropical house crickets [32] and ants [22]. Nonetheless, the available data show that different insect species respond differently to M/NPs because of the fact that M/NPs vary considerably in terms of particle size, shape, concentration and exposure time [6,19,30,32]. In addition, plastic polymers can pose significant risks to insects at molecular, cellular and organismal levels [6,8]. Thus, the effects of M/NPs on the ecology and physiology of terrestrial insects have been insufficiently examined so far, highlighting that more empirical studies are needed to elucidate the mechanisms and cause–effect relationship of M/NPs on insects.

Ants are a prominent eusocial insect species in almost all terrestrial ecosystems worldwide [33]. They make up at least one-third of the total insect biomass and deliver essential services, including biological control, plant pollination, soil regeneration, nutrient cycling, seed dispersal and bioindication [34,35]. Previous studies have confirmed that the ant colonies are perennial and wingless workers ants could actively transport M/NPs to different soil horizons [36,37]. Such transport by ants may expand the distribution of M/NPs and influence the exposure of other soil fauna to M/NPs in soil ecosystems [38]. Meanwhile, M/NPs may accumulate in their bodies with adverse effects on fitness at individual and colony levels [39]. Thus, the transport and accumulation of nature toward M/NPs by ants make them a good object for studying the impact of M/NPs contaminants on terrestrial insects [40]. However, to our knowledge, only a few studies have revealed the effects of M/NPs on food choice [40], the transport capacity of M/NPs [38] and biological traits of the queen [39]. Currently, researchers lack more knowledge of whether and to what extent M/NPs affect this eusocial insect species.

The Japanese carpenter ant, *Camponotus japonicus* Mayr (Hymenoptera: Formicidae), is one of the most common and widespread ant species in China and other countries in East Asia [41]. This ant species is a natural enemy for many forestry and agricultural pests as well as one of the useful medical insects [42]. In addition, this species is usually polyphagous, cooperative and sometimes with polymorphic worker forces, allowing them to use a wide range of prey types [43]. A mature nest of this species can have up to three queens with colonies that can reach a colony size of over 4000 workers [44]. The foraging and nesting activity is evidently a vital task exhibited by worker ants [45]. The pervasive nature of M/NPs makes the contact between M/NPs and ants inevitable, thereby exposing them to the risk of a decline in biological traits. However, no data are available about the potential effects of M/NPs on the biological traits of this ant species. As a result, there is a pressing need to bridge this knowledge gap to gain a better understanding of the effects of M/NPs on this ant species.

In the present study, we used the worker ants *C. japonicus* as model insects and polystyrene microplastic (PS-MP)- and polystyrene nanoplastic (PS-NP)-contaminated honey aqueous solutions containing three particle sizes (0.05, 1 and 50 μm) and four concentrations (0.1, 1, 10, 50 mg/mL) as testing materials to evaluate the potential impact of these contaminants on the biological traits of ants in general under laboratory conditions in order to test the hypothesis that dietary exposures of PS-M/NPs can incur adverse effects in terms of foraging behavior, food consumption, digging ability, body weight and survival in this ant species, and these adverse effects may be regulated by the particle size, concentration and exposure time of PS-M/NPs. For this purpose, we conducted three experiments as follows. First, we assessed the foraging preference and food consumption of worker ants when providing PS-M/NP-contaminated solutions and non-contaminated solutions simultaneously or separately to them. Second, we elucidated the potential impact of PS-M/NPs on the digging ability of worker ants after the ingestion of PS-M/NP-contaminated solutions on different days. Finally, we measured the changes of body weight and survival of worker ants after fed with PS-M/NP-contaminated solutions at different particle size and concentration levels. These results will provide an effective way to evaluate the potential impact of PS-M/NPs and could contribute to filling the gap in the knowledge of these ubiquitous contaminants on this ant species.

## 2. Materials and Methods

### 2.1. Collection and Housing of C. japonicus

Three field colonies of the Japanese carpenter ant *C. japonicus* were collected from the woodland (E 117°21′11.88″, N 27°54′54.36″) of Guixi, Jiangxi Province, China, in May 2022. We transferred each colony into a plastic case with Fluon coated on the inner walls using a shovel, then the ants were removed from primitive nests using the water floating method and housed each colony in a transparent plastic box (length × width × height = 37 × 25 × 11 cm^3^). At the bottom of each rearing box, we provided twenty opaque glass test tubes (diameter = 1.8 cm, height = 18 cm) as nests, one inverted transparent plastic bottle (d = 3.5 cm, h = 5.5 cm) with tapped water plugged using a cotton ball to maintain constant humidity and one plastic feeding tray (d = 2 cm, h = 0.5 cm) with 1:3 (*v*/*v*) mixture of honey and distilled water as food for this ant species. Each colony had approximately 1000 worker ants with a queen and brood. Fresh pupae of super mealworm *Zophobas morio* were provided as a food supplement for animal protein. The rearing boxes were maintained in a constant incubator at 25 ± 1 °C and relative humidity of 60 ± 5% with a photoperiod of 12:12 (light:dark) h. Food and water were replaced once every 2 days to maintain the normal activities of the ants. Field-collected ants were considered to have acclimatized to the laboratory environment when queens in each colony began to lay eggs. Then, the worker ants of each colony were selected and used for the following experiments.

### 2.2. Preparation of PS-M/NPs Food Media

Polystyrene microplastics (PS-MPs) and polystyrene nanoplastics (PS-NPs) were chosen in this study because they are among the most abundant polymers in the environment, and their increasing global production is expected to reach 15.68 million metric tons by 2024 [30]. In the current study, we consulted the method proposed by Deng et al. [28], and a commercial polystyrene microsphere with three particle sizes (d = 0.05, 1 and 50 μm, respectively) was purchased from Haian Zhichuan Battery Materials Technology Co., Ltd., Nantong, China. These particles were red fluorescent monodispersed and supplied in 20 mL milk-white aqueous suspensions with a solid content of 10% (*w*/*v*). These plastic materials were then stored at ambient temperature before use.

Prior to the experiment, aqueous honey solutions containing polystyrene microspheres with three particle sizes and four concentration levels were prepared as food media for experimental worker ants. The following general procedure was improved and applied according to the methods of Balzani et al. [6] and Tang et al. [46]. A total of 50 mL of honey was measured using a measuring cylinder and then transferred into a small glass beaker with 150 mL of distilled water to dilute up to 200 mL of aqueous honey solution. This aqueous honey solution was utilized as a non-contaminated aqueous honey solution in subsequent experiments, as well as a diluent solution for PS-M/NP-contaminated aqueous honey solutions. Subsequently, 0.01, 0.10, 1.00 and 5.00 mL of polystyrene microsphere (10% *w*/*v*) with a size of 0.05 μm were transferred into four glass tubes with 9.99, 9.90, 9.00 and 5.00 mL of non-contaminated aqueous honey solution using glass capillary pipette puller, respectively. Thus, we finally obtained 10 mL of PS-M/NP-contaminated honey aqueous solution with a size of 0.05 μm at concentrations of 0.1, 1, 10 and 50 mg/mL, respectively. By using the same preparation procedure, we separately obtained 10 mL of PS-M/NP-contaminated honey aqueous solution with particle sizes of 1 μm and 50 μm at four different concentrations. In this study, twelve PS-M/NP-contaminated honey aqueous solutions and one non-contaminated honey aqueous solution were prepared as food media for the following experiments. To make the PS-M/NPs uniformly distributed in the aqueous honey solutions, all of the prepared aqueous honey solutions were shaken well using a vortex oscillator before use.

### 2.3. Effect of PS-M/NPs on Foraging Behavior of Worker Ants

To evaluate the impact of PS-M/NPs on the foraging behavior of work ants *C. japonicus*, we performed multiple-choice trial and no-choice trial, respectively, according to the methods reported by Cuvillier-Hot et al. [16] and El Kholy and Al Naggar [19]. In the multiple-choice trial, three particle size groups (0.05, 1 and 50 μm) and four concentration groups (0.1, 1, 10 and 50 mg/mL) were carried out. Before bioassay, five plastic feeding trays were placed evenly at random in a small plastic Petri dish (d = 8.5 cm, h = 1.5 cm) in each particle size group, and each feeding tray was poured 1 mL of PS-M/NP-contaminated solution at a concentration of 0 (non-contaminated solution, control), 0.1, 1, 10 and 50 mg/mL, respectively. Similarly, in each concentration group, four plastic feeding trays were placed evenly at random in a small plastic Petri dish, and each feeding tray was poured 1 mL of PS-M/NP-contaminated solution at a particle size of 0 (control), 0.05, 1 and 50 μm, respectively. In the no-choice trial, one feed tray containing 1 mL of a designated PS-M/NP-contaminated solution or non-contaminated solution was placed in a Petri dish.

During bioassay, a single 24 h-starved worker ant was then selected from any of three experimental colonies and introduced into the Petri dish containing food media. Subsequently, the foraging behavior of each worker ant was monitored for 2 min using a digital video camera (high definition, 720 P, 1/2.7″ Sony CCD camera, Tokyo, Japan). After finishing the monitoring schedule, we checked each video and recorded the behavioral parameters of each experimental worker ant, including the pre-foraging period (as the time elapsed from the introduction of the worker ants into the Petri dishes to their first visitation of aqueous honey solution), percentage of licking (shown by the proportion of licking frequency in visiting frequency) and food consumption (recorded by measuring the weight of the plastic feeding tray before and after each experiment using electronic balance). In addition, the food media type that was firstly chosen by each worker ant was recorded, and the percentage of choice were analyzed in multiple-choice trials.

Behavioral observations were conducted in an air-conditioned chamber at 25 ± 1 °C and a relative humidity of 60 ± 5% with a photoperiod of 12:12 (light:dark) h. All experimental individuals were used only once in this experiment. In each of the three particle size group and four concentration group, ten worker ants from each of three colonies (i.e., thirty repetitions) were performed. In the no-choice trial, we performed twelve PS-M/NP-contaminated solution treatments and one non-contaminated solution treatment, with three repetitions per treatment and five worker ants per repetition.

### 2.4. Effect of PS-M/NPs on Digging Ability of Worker Ants

In this experiment, a self-designed digging arena consisting of a Petri dish (d = 8.5 cm, h = 1.5 cm) and a small plastic tube with a lid (d = 1.8 cm, h = 18 cm) was established following the method described by Chen [47]. The lid of the small plastic tube was connected to the bottom center of the Petri dish using hot melt adhesive, and a 3-mm access hole was drilled through the center of the Petri dish and the lid underneath it. The treated fine river sands with the same particle sizes were used as available digging substrates. Prior to the start of this experiment, the collected sands were rinsed with clean water to eliminate impurities and then sieved in succession using a mesh size of 0.33 mm and 0.30 mm in sequence. Then, the sands with 0.28 ± 0.02 mm in diameter were selected and dried and sterilized in an oven at 150 °C for 12 h to eliminate aerobic microorganisms. Subsequently, the treated fine sands were adjusted the moisture content to 12% by adding 26.08 mL of water into 200 g of sand in the beaker and stirring the sand with a glass rod for 2 min to ensure mixing of water with sand. The fine sands with adjusted moisture content were filled into the small plastic tube. The lid of the small plastic tube was covered, and the inside wall of the Petri dish was coated with Fluon to prevent the experimental ants from escaping.

To evaluate the digging ability of worker ants *C. japonicus* after the ingestion of PS-M/NP-contaminated solutions, the worker ants with similar body sizes were selected from any of three experimental colonies and introduced into the Petri dishes at a density of 10 individuals per rearing container. We reared these experimental individuals for 28 d using twelve PS-M/NP-contaminated solutions and one non-contaminated solution as food media, respectively. The digging ability of worker ants that ingested different solutions was observed on days 0, 7, 14, 21 and 28 in the self-designed digging arena, respectively. At the beginning of the bioassay, one worker ant that ingested one of the food media was introduced into one prepared digging arena and permitted a 24 h free activity in an air-conditioned chamber under the same ambient conditions described above. After finishing the bioassay, the experimental individuals were removed from the digging arena. The fine sands in the bottom of each Petri dish were then collected and dried in an oven at 150 °C for 4 h. Thereafter, the weights of the fine sands were measured using an electronic balance (sensitivity 0.1 mg). In this experiment, twelve experimental treatments and one control treatment were conducted, and thirty worker ants were carried out for each treatment. All experimental individuals were used only once.

### 2.5. Effect of PS-M/NPs on Body Weight and Survival of Worker Ants

To assess the potential effects of PS-M/NPs on body weight and survival of the carpenter ant *C. japonicus*, we randomly selected sixty worker ants from each of the three experimental colonies and transferred them into the bigger Petri dishes (d = 20 cm, h = 2 cm) at a density of thirty individuals per Petri dish. Then, the experimental worker ants in each Petri dish were provided with one designated food media. The experimental individuals were kept in a constant incubator under the same ambient conditions described above. Food media in each Petri dish were replaced once every 2 days until the experiment ended. The number of dead work ants and the body weight of the alive worker ants were then recorded on days 0, 7, 14, 21, 28, 35 and 42. In this experiment, twelve PS-M/NP-contaminated solutions and one non-contaminated solution were used as treatments. For each food treatment, we carried out six repetitions, and thirty worker ants were adopted for each repetition.

### 2.6. Data Analysis

The statistical analyses were carried out with SPSS version 25.0 for Windows (SPSS Inc., Chicago, IL, USA). All data were checked for normality using the Kolmogorov–Smirnov test, and Levene’s test was employed to determine the homogeneity of error variance. The body weight data were log_10_-transformed to better approximate normality and homogeneity of variances. The percentage data were subjected to arcsine square root transformation prior to analysis. Data that followed the normal distribution (*p* > 0.05) were treated in parametric tests, while non-normal data (*p* < 0.05) were evaluated with non-parametric statistical tools. In the multiple-choice trial, the difference in foraging behavior and food consumption was assessed using a one-way ANOVA followed by Tukey’s HSD test or a non-parametric Kruskal–Wallis test if the data passed or failed the normality of distribution and homogeneity of variances. The possible effects of PS-M/NPs on the foraging behavior of experimental work ants in the no-choice trial were investigated by means of a generalized linear model (GLM) with pre-foraging period, percentage of licking and food consumption as the dependent variable, respectively, and particle size and concentration of foods as fixed factors. The variation of digging ability and body weight of experimental worker ants were also subjected to GLM with the weight of fine sands and body weight as dependent variables, respectively, and particle size, concentration of foods and exposure time as fixed factors. To detect differences between the different groups, we used Tukey’s post hoc tests. A comparison of the survival rate among treatments was performed using Kaplan–Meier estimation and log-rank tests. The significant level adopted was equal to or lower than 0.05. All data are presented as means ± standard errors (SEs).

## 3. Results

### 3.1. Effect of PS-M/NPs on Foraging Behavior in Multiple-Choice Trial

**Figure 1:**
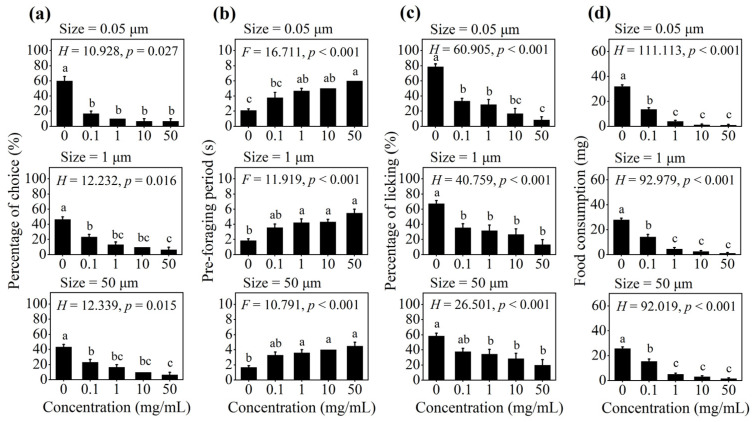
The effect of concentration of PS-M/NPs on the foraging behavior of *C. japonicus* in the multiple-choice trial. (**a**) percentage of choice; (**b**) pre-foraging period; (**c**) percentage of licking; and (**d**) food consumption. The different lowercase letters above each bar indicate significant difference (one-way ANOVA followed by Tukey’s HSD test or a non-parametric Kruskal–Wallis test, *p* < 0.05).

For each particle size group, when given a choice among the non-contaminated solution and four PS-M/NP-contaminated solutions with different concentrations, over 43.3% of worker ants showed significant foraging preference for the non-contaminated solution, and the percentage of choice decreased with increasing concentration of PS-M/NPs (*p* < 0.05; Figure 1a). The pre-foraging period of worker ants was prolonged with the increase in the concentration of PS-M/NPs, and significant differences were found in each particle size group (*p* < 0.01, Figure 1b). Regardless of particle size, the percentage of licking of worker ants was the highest when exposed to the non-contaminated solution but showed a downward trend with increasing concentration (*p* < 0.01; Figure 1c). Furthermore, worker ants fed on non-contaminated solutions showed the highest food consumption compared with PS-M/NP-contaminated solutions at different concentrations (*p* < 0.001, Figure 1d).

**Figure 2:**
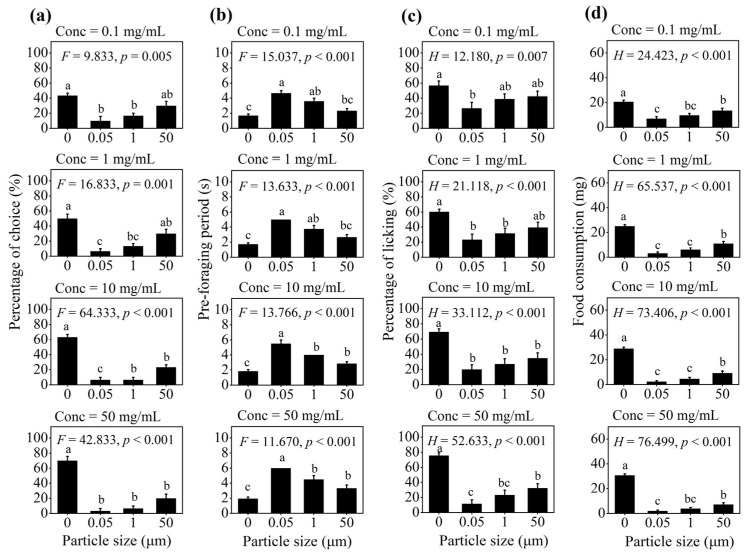
The effect of particle size of PS-M/NPs on the foraging behavior of *C. japonicus* in the multiple-choice trial. (**a**) percentage of choice; (**b**) pre-foraging period; (**c**) percentage of licking; and (**d**) food consumption. The different lowercase letters above each bar indicate significant difference (one-way ANOVA followed by Tukey’s HSD test or a non-parametric Kruskal–Wallis test, *p* < 0.05).

For each concentration group, worker ants more often (over 43.3%) chose non-contaminated solutions, while less than 30% of individuals chose PS-M/NP-contaminated solutions with different particle sizes (*p* < 0.01; Figure 2a). The longest pre-foraging period was recorded for the solution with 0.05 μm of particle size compared with the 1 and 50 μm ones and non-contaminated solution (*p* < 0.001, Figure 2b). With an increase in the particle sizes from 0.05 μm to 50 μm in four concentration groups, the percentage of licking of worker ants increased by 1.59, 1.69, 1.75 and 2.79 times, and the food consumption of worker ants increased by 1.93, 3.44, 3.78 and 3.57 times, respectively, but all were substantially lower than those fed on the non-contaminated solution (all *p* < 0.01, Figure 2c,d).

### 3.2. Effect of PS-M/NPs on Foraging Behavior in No-Choice Trial

**Figure 3:**
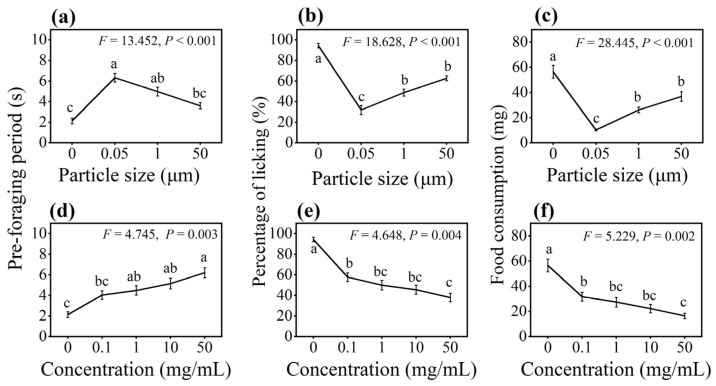
The effect of particle size (**a**–**c**) and concentration (**d**–**f**) of PS-M/NPs on the foraging behavior of *C. japonicus* in the no-choice trial. (**a**,**d**) pre-foraging period; (**b**,**e**) percentage of licking; and (**c**,**f**) food consumption. The different lowercase letters above each data point indicate significant difference (GLM and Tukey’s post hoc tests, *p* < 0.05).

In the no-choice trial, worker ants that were fed on of the non-contaminated solution showed the shortest pre-foraging period and the highest percentage of licking and the highest food consumption compared with those that were fed on PS-M/NP-contaminated solutions. GLM analysis revealed that the foraging behavior of worker ants was influenced either by the particle size or by the concentration of PS-M/NPs (Figure 3). As the particle size increased from 0.05 μm to 50 μm, the pre-foraging period was significantly shortened by 1.76 times (GLM, *p* < 0.001, Figure 3a), while the percentage of licking and food consumption significantly increased by 1.97 times (*p* < 0.001, Figure 3b) and 3.58 times (*p* < 0.001, Figure 3c), respectively. As the concentration increased from 0.1 to 50 mg/mL, the pre-foraging period significantly prolonged by 1.53 times (*p* = 0.003, Figure 3d), while the percentage of licking and food consumption was significantly decreased by 1.52 times (*p* = 0.004, Figure 3e) and 1.93 times (*p* = 0.002, Figure 3f), respectively.

### 3.3. Effect of PS-M/NPs on Digging Ability of Worker Ants

**Figure 4:**
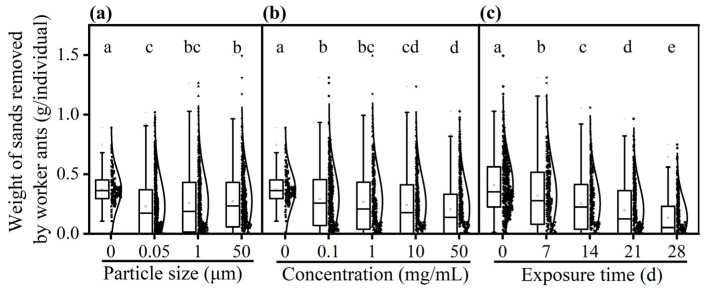
The effect of particle size (**a**), concentration (**b**), and exposure time (**c**) on the digging ability of worker ants *C. japonicus* when fed on non-contaminated and PS-M/NP-contaminated food media. The different lowercase letters above each data point indicate significant difference (GLM and Tukey’s post hoc tests, *p* < 0.05).

The weight of sand removed by worker ants in the non-contaminated solution treatment was significantly heavier than those in PS-M/NP-contaminated solution treatments (Figure 4). GLM analysis revealed that the weight of sand removed by worker ants was influenced by the particle size, concentration, and exposure time of PS-M/NPs. The worker ants that were fed on honey aqueous solution spiked with 0.05 μm of PS-NPs significantly removed less sand compared with those fed on non-contaminated honey aqueous solution (control) and those fed on greater particle size of PS-MPs (*F* = 6.155, *df* = 3, 1950, *p* = 0.002, Figure 4a). The weight of sand removed by the worker ants was the lightest (0.20 ± 0.01 g/individual) when it was fed on the PS-M/NP-contaminated solution with the highest concentration (50 mg/mL) and the heaviest (0.37 ± 0.01 g/individual) when it was fed on the non-contaminated solution (*F* = 13.037, *df* = 4, 1950, *p* < 0.001, Figure 4b). As the exposure time to PS-M/NPs extended, the weight of the sand removed by worker ants showed a significant downward trend (*F* = 65.984, *df* = 4, 1950, *p* < 0.001, Figure 4c).

### 3.4. Effect of PS-M/NPs on Body Weight of Worker Ants

**Figure 5:**
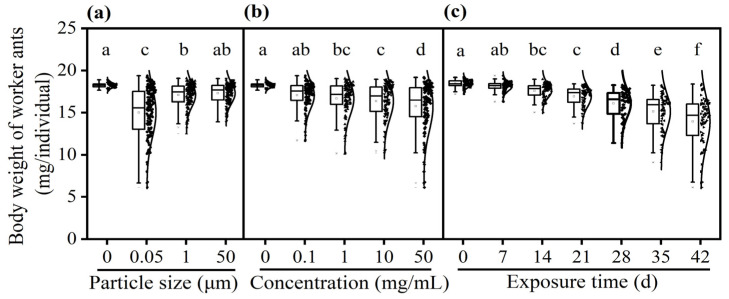
The effect of particle size (**a**), concentration (**b**) and exposure time (**c**) on the body weight of worker ants *C. japonicus* when fed on non-contaminated and PS-M/NP-contaminated food media. The different lowercase letters above each data point indicate significant difference (GLM and Tukey’s post hoc tests, *p* < 0.05).

The body weight of worker ants varied across different food media treatments. The worker ants that fed on non-contaminated solutions had the heaviest body weight compared with individuals fed on PS-M/NP-contaminated solutions (Figure 5). Worker ants experienced greater weight loss when exposed to PS-M/NP-contaminated solutions with smaller particle size (*F* = 60.682, *df* = 3, 546, *p* < 0.001, Figure 5a) or with higher concentration (*F* = 49.835, *df* = 4, 546, *p* < 0.001, Figure 5b). In addition, the body weight of worker ants showed a significant downward trend when the exposure time of worker ants to PS-M/NP-contaminated solutions was extended (*F* = 86.745, *df* = 6, 546, *p* < 0.001, Figure 5c).

### 3.5. Effect of PS-M/NPs on the Survival of Worker Ants

**Figure 6:**
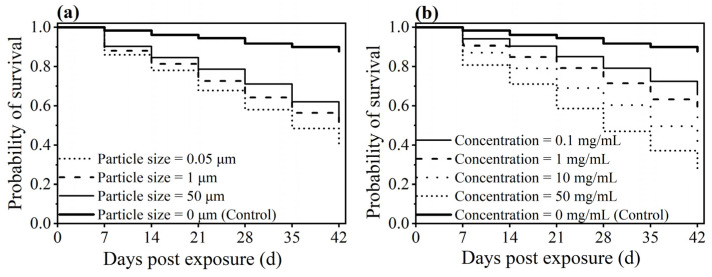
The effect of particle size (**a**) and concentration (**b**) on the survival of worker ants *C. japonicus* when fed on non-contaminated and PS-M/NP-contaminated food media.

The presence of PS-N/MPs in the food media significantly affected the survival of the worker ants. After 42 days of exposure to PS-N/MP-contaminated solutions, the survival rates of worker ants were 38.47%, 47.92% and 54.31% for the 0.05, 1 and 50 μm of particle size treatments, respectively, showing values significantly lower than the control (87.78%) (log-rank (Mantel–Cox) test, *χ^2^* = 129.699, *df* = 3, *p* < 0.001, Figure 6a). The survival rates of worker ants were 65.74%, 54.07%, 39.63% and 28.15% for the 0.1, 1, 10 and 50 mg/mL of concentration treatments, respectively, all of which were significantly lower than the control (*χ^2^* = 299.897, *df* = 4, *p* < 0.001, Figure 6b).

## 4. Discussion

In the present study, we investigated the potential impact of PS-M/NPs on the Japanese carpenter ant *C. japonicus* by examining certain biological traits under laboratory conditions. Three clear findings were generated from our study. First, PS-M/NPs affected the foraging behavior of worker ants, showing that the smallest particle size and the highest concentration of PS-M/NPs resulted in the lowest percentage of choice, longest pre-foraging period, and lowest percentage of licking and the amount of food consumption. Second, PS-M/NPs affected the digging ability of worker ants, exhibiting that the weight of sand removed by worker ants decreased significantly with decreasing the particle size, increasing the concentration and prolonging the exposure time of PS-M/NP-contaminated solutions. Finally, the body weight and survival of worker ants showed a dramatic decline as the particle size decreased, the concentration increased and the exposure time prolonged in PS-M/NP-contaminated solutions. The results of this study confirm the initial hypothesis that PS-M/NPs mixed in food media have an adverse effect on the Japanese carpenter ant *C. japonicus*. Small particle size, high concentration and longer exposure time are the key factors in decreasing the foraging behavior, digging ability, body weight and survival of this ant species.

Previous studies have shown that insect behavior, including predacious behavior, locomotor behavior, foraging behavior and walking behavior, could be altered using M/NPs during trophic transfer [6,23,48,49]. Some insects, such as the black garden ant *Lasius niger* [16], the black fungus gnat *Bradysia difformis* [50] and the fruit fly *Drosophila melanogaster* [19], show the ability to detect and avoid feeding on food contaminated with M/NPs or prefer habitats free of M/NPs. Some insects, such as the non-biting midge larvae *Chaoborus flavicans* [51] and the honey bee *Apis mellifera* [7], do not show distinct avoidance or preference. Nevertheless, some insects, such as the acrobat ant *Crematogaster scutellaris*, exhibit foraging preferences in the early stage of exposure to food but not in the later stage [40]. Our results obtained via a multiple-choice experiment showed that the worker ants *C. japonicus* more often prefer to choose non-contaminated solutions rather than PS-M/NP-contaminated solutions, regardless of particle size and concentration of M/NPs. Meanwhile, worker ants consumed non-contaminated solutions faster, more frequently and in higher quantities than PS-M/NP-contaminated solutions in the multiple-choice trial and in the no-choice trial. These results demonstrate that the worker ants *C. japonicus* also show the ability to distinguish between non-contaminated solutions and PS-M/NP-contaminated solutions and chase the former. A recent study revealed that the plastic particles applied do not have any taste, but ants can detect the presence of solid particles in a solution [52]. Thus, the M/NPs detection by ants might be due to the significant accumulation of M/NPs in their mouths. In addition, solutions contaminated with PS-M/NPs might have a repellent effect on ant feeding, as it increases the viscosity of the solution. This possible reason has been confirmed in the honeybee *A. mellifera* [10] and the acrobat ant *C. scutellaris* [40].

Current data found that PS-M/NPs affect the foraging behavior of worker ants in a particle size and concentration-dependent manner; the smaller the size and higher the concentration of the M/NPs, the greater the adverse effects. Similar results have been reported in other insects [6]. The reason for this result may be related to the fact that worker ants are more efficient in taking up less viscous solutions. Increasing concentrations of M/NPs will make for more viscous solutions, leading to a repellent effect for the foraging behavior of worker ants. Meanwhile, exposing work ants to smaller particles of PS-MPs would block their alimentary canal, destroy their midgut tissue and translocate to tissues and organs. Our findings are consistent with earlier studies conducted with the honeybee *A. mellifera* [7,28] and the black fungus gnat *B. difformis* [50]. Future studies are needed to further understand the recognition mechanisms of M/NPs by this ant species.

Digging behavior is critical to ant species since digging is one of the fundamental behaviors involved in building nests and foraging tunnels [53]. In the present study, all of the worker ants had the capability to dig the treated fine sands, regardless of ingesting non-contaminated solutions or PS-M/NP-contaminated solutions. However, the weight of sand removed by worker ants was significantly reduced when exposed to PS-M/NP-contaminated solutions compared with non-contaminated solutions. Significant differences in the weight of sands might be explained by the sublethal adverse effects of the PS-M/NPs. Furthermore, we found that the weight of sand removed by worker ants was statistically reduced with the increase in particle size, the decrease in concentration and the prolonging of the exposure time of PS-M/NP-contaminated solutions. This finding suggests that the adverse effect of PS-M/NPs was also influenced by particle size, concentration and exposure time. One possible explanation is that worker ants exposed to smaller particles, higher concentrations of PS-M/NPs and lasting for a longer time might have been significantly weakened, resulting in a significantly lower weight of sand being removed from the arena. A similar phenomenon was described in the red imported fire ant *Solenopsis Invicta* [47].

Most terrestrial insects have reported that the exposure of various insect species to M/NPs may reduce their body weight and impede their survival [30]. However, such adverse effects are also regulated by multiple factors of M/NPs, such as particle size, concentration and exposure time. For instance, the tropical house cricket *Gryllodes sigillatus* grown on PET-MP-treated food exhibited significantly lower adult body weight compared with control individuals fed “clean” food [32]. The weight of the black soldier fly *Hermetia illucens* larvae grown on food waste containing PE was lower than that of flies in the control on day 6 but was exceeded on day 20 and on day 24 [54]. In honeybee *A. mellifera*, a significant negative effect of M/NPs on body weight and survival was found with the ingestion of small-sized and higher-concentration PS-MPs [6,29,31]. In contrast, the effect of M/NPs on the survival of insects varied with exposure time. For example, chronic exposure to PS-MP fragments has no effect on the survival of honeybee *A. mellifera* [29], while *D. melanogaster* fly survival was significantly lower than controls after 7 days of chronic exposure to PS-MP concentrated food media [19]. Based on current observations of the Japanese carpenter ant *C. japonicus*, worker ants fed on non-contaminated solutions had the heaviest body weight and highest survival compared with individuals fed on PS-M/NP-contaminated solutions. In addition, the body weight and the probability of survival were significantly reduced by decreasing the particle size, increasing the concentration and extending the exposure time of the PS-M/NPs. These data further suggest that the small size, high concentration and long-term exposure time of the PS-M/NPs are normally associated with high toxicity potential for this insect. The possible reason for this is that NPs are more likely to enter cells and tissues and thus have more widespread effects on the organism than MPs, as reported in the study of the non-biting midge *Chironomus riparius* [55] and *D. melanogaster* [19]. In addition, high concentration and long-term exposure of PS-M/NPs might be related to the disruption of the gut microbiota by M/NPs or its direct physical impact on the host gut, leading to necrosis and apoptosis of intestinal epithelial cells; in particular, PS-NPs might suppress the immune response and the hosts exhibit higher mortality after infection. These results are consistent with previous studies on other insects [19,30]. However, this hypothesis would need to be validated by gut microbiota analysis of this insect species.

## 5. Conclusions

The current study confirms that dietary exposures to PS-M/NPs cause adverse effects on food choice, food consumption, digging ability, body weight and survival in the worker ants *C. japonicus*. However, this adverse effect of PS-M/NPs is regulated by its particle size, concentration and exposure time, with small size, high concentration and long-term exposure of the PS-M/NPs having high toxicity potential for this ant species. Our findings provide a baseline understanding of the effects of PS-M/NPs on worker ants and give a warning that management measures against PS-M/NPs must be taken. Future studies should assess the effects of multi-generational exposure to PS-N/MPs and explore the potential mechanisms by transcriptomic analysis. In addition, the adverse effects of M/NPs on the biological traits of ants may lead to certain secondary consequences, such as a decrease in the pest control of this species. This speculation also needs further investigation.

## Data Availability

All data generated and/or analyzed during the current study are available from the corresponding author upon request.
